# Magnetic resonance myocardial perfusion imaging in the diagnosis of functionally significant obstructive coronary artery disease: a systematic review protocol

**DOI:** 10.1186/2046-4053-3-53

**Published:** 2014-05-26

**Authors:** Rebabonye B Pharithi, Martinus Meela, Thomas Kropmans, Frank Ward, Michael Conway, Michael Newell

**Affiliations:** 1 Department of Cardiology, St Lukes hospital, Freshford Road, Kilkenny, Republic of Ireland; 2 Department of Anaesthesia, Children University Hospital, Temple Street, Dublin 1, Republic of Ireland; 3 Department of Medical Informatics and Medical Education, National University of Ireland, Galway, Republic of Ireland

**Keywords:** Cardiac magnetic resonance, Fractional flow reserve, CMR, FFR, Quality assessment of diagnostic accuracy studies 2 tool, QUADAS2, STATA 13, Coronary artery disease, Meta-analyses and systematic review protocol

## Abstract

**Background:**

Cardiac magnetic resonance (CMR) myocardial perfusion imaging has been suggested as a non-invasive alternative to pressure wire guided fractional flow reserve (FFR) in detecting haemodynamically significant obstructive coronary artery disease (CAD). The objective of this systematic review is to determine the diagnostic accuracy of CMR and to compare it to FFR.

**Methods/design:**

A systematic review of diagnostic test accuracy of CMR and FFR will be conducted. Relevant English-language articles published before November 2013 in Medline, PubMed, EMBASE, Google scholar, Scopus and Cochrane databases will be identified. Relevant referenced articles from those selected will also be analysed. Articles describing diagnostic studies that compared CMR to FFR in patients with known or suspected coronary artery disease will be included. Two investigators will independently screen, assess quality and extract data from the selected articles. The Quality Assessment of Diagnostic Accuracy Studies 2 (QUADAS-2) tool will be used to assess methodological quality. STATA 13 (the xtmelogit command) software will be used to calculate bivariate random effects models and estimate pooled sensitivity and specificity with 95% confidence intervals. Forests plots and summary receiver operating characteristics curves will also be generated. Sub-group pooled analyses using image quality of CMR (in terms of magnetic flux density - *Tesla*) and basis of analyses (coronary arterial territory *vs.* patients basis) at different FFR cutoffs (≤0.75 and ≤0.8) will also be performed.

**Discussion:**

This systematic review will help to determine if CMR is an adequate alternative to FFR in the diagnosis of significant and obstructive CAD. We will also be able to assess diagnostic accuracy of specific types of CMR scan at different FFR cutoffs.

**Systematic review registration:**

This systematic review had been registered at PROSPERO and the registration number is CRD42013006180.

## Background

Ischaemic heart disease (IHD) as a consequence of untreated coronary artery disease (CAD) is one of the top three causes of mortality and the most common cause of morbidity in developed countries [1,2]. Therefore, early diagnosis is important. Assessment of haemodynamically significant narrowing of coronary arteries with pressure-wire guided fractional flow reserve (FFR) is a widely accepted practice by interventional cardiologists [3-6]. However, this technique is invasive and exposes patients to ionizing radiation. Hence, the use of non-invasive diagnostic tools with less or no radiation is desirable.

Cardiac magnetic resonance (CMR) perfusion imaging is a well-established and non-invasive technique that has been shown to be safe in the diagnosis of obstructive coronary lesions [7]. In a single exam, both anatomical and functional information can be integrated with high spatial and temporal resolution without exposing the patient to harmful radiation [8].

Several small individual studies have evaluated the diagnostic performance of CMR compared to FFR, with its diagnostic power varying among reports. Some of these studies have been included in a meta-analyses which compared CMR to quantitative coronary angiography (QCA) [9]. As several reports have been published after the above meta-analyses [10-13], and also owing to the fact that QCA often provides insufficient information regarding physiological significance of the coronary lesions, an updated systematic review of CMR diagnostic accuracy compared with an invasive pressure-wire guided FFR is required.

The primary objective is to assess the diagnostic accuracy of CMR perfusion imaging in the detection of functionally significant, obstructive coronary artery lesions in patients with known or suspected CAD, in comparison to FFR.

The secondary objective is to assess the potentially relevant-patient benefit (prognosis) of CMR perfusion imaging in the diagnosis of haemodynamically significant, obstructive but stable CAD. We also plan to investigate the diagnostic accuracy of CMR on the basis of magnetic flux density (in Tesla) and basis of analysis (coronary artery territory *vs.* patient analysis) and the methodological quality of the included studies.

## Methods/Design

### Design

The design of this systematic review follows the methodological approach as recommended by the Cochrane collaboration methods group on screening and diagnostic tests [14], the Agency for Healthcare Research and Quality and the United Kingdom National Institute for Health and Clinical Excellence. This protocol was written in accordance with the preferred reporting items for systematic review and meta-analysis (PRISMA) [15]. This protocol has also been registered with PROSPERO (#CRD42013006180).

### Search strategy

Relevant original English-language articles published prior to November 2013 in OVID Medline, Cochrane, Embase, Scopus, Google Scholar and PubMed databases will be electronically searched. Reference lists of all the retrieved papers will also be extensively cross-checked to supplement the list of the selected articles. Details of the specific search strategies for the relevant databases are shown in Additional file 1 (search strategy for OVID Medline database) and Additional file 2 (search strategy for PubMed Database).

### Eligibility criteria and study selection

Search terms are present either in the title or abstract for the articles to be considered for analysis. Studies selected for inclusion are those which evaluate the diagnostic accuracy of CMR perfusion imaging (index test) for the detection of functionally significant coronary artery lesions (target condition) compared to an appropriate reference standard. The reference standard which will be used is coronary angiogram guided pressure wire-derived FFR. An FFR cutoff of either <0.75 or ≤0.80 will be used. These cutoffs are used because they include the narrow ‘grey zone’ (0.75-0.80) associated with inducible myocardial ischaemia with accuracy of ≤0.75 being above 90% [16]. An FFR cutoff of ≤0.80 has widely been used in multi-vessel CAD since its introduction by the FAME investigators [17]. The two cutoffs are still used interchangeably by researchers at this point.

Other forms of reference standard, such as single photon emission computed tomography (SPECT), visual coronary angiogram alone, stress echocardiogram and electrocardiogram or computed tomography coronary angiogram, will not be included so as to avoid verification bias [18]. Two reviewers will independently screen the titles and the abstracts to deem a study suitable for inclusion. Subsequently, the entire manuscript will be assessed by one reviewer and double checked by another. Disagreements will be resolved by consensus or referral to a third reviewer. Review articles (to avoid repeated data), unpublished studies, letters, comments and case reports will be excluded.

### Data selection

Eligible articles, fulfilling our inclusion criteria, will be independently reviewed by two authors. The data to be extracted will include the author, country, year of publication, number and age of subjects, type of study, either single or multi-vessel CAD, diagnostic test characteristics and threshold for critical functional coronary arterial luminal stenosis (FFR ≤0.75 and or ≤0.8). Where values are not available we will also manually extract and calculate values for true positive (TP), false positive (FP), true negative (TN), false negative (FN), sensitivity, specificity, accuracy, positive predictive value, negative predictive value, positive likelihood ratio and negative likelihood ratio results in the detection of haemodynamically significant obstructive coronary artery lesions. Data for the diagnostic performance of CMR perfusion imaging against FFR will be displayed in contingency 2 × 2 tables. In order to allow the calculation of outcomes measures of interest, empty cells will be filled with 0.5 of events.

### Assessment of methodological quality

Two reviewers will independently assess methodological quality of included studies by using the published QUADAS-2 (the Quality Assessment of Diagnostic Accuracy studies) tool [19]. CMR results should be interpreted without knowledge of FFR. The QUADAS-2 tool was chosen because it assesses the risk of bias by methodological domain (participant selection, index test, reference standard and flow of participant through the study (see Additional file 3) rather than the more general overall risk of bias - as evident in the original QUADAS. The agreement or disagreement between the reviewers (inter-rater reliability) in evaluating methodological quality will be calculated with Cohen Kappa coefficient (*k*) [20]. The items in QUADAS-2 will be as recommended by the Cochrane collaboration methods group on screening and diagnostic tests [14], the Agency for Healthcare Research and Quality and the United Kingdom National Institute for Health and Clinical Excellence. This tool will be applied in four phases: (1) the review question will be summarised; (2) the tool will be tailored to produce a review-specific guidance; (3) a flow diagram for the primary study will be constructed; and finally (4) bias and applicability will be judged. See Additional file 3 which shows an adapted QUADAS-2 in assessment of the risk of bias and applicability.

### Outcomes

The primary outcome of this systematic review is to assess the diagnostic accuracy of CMR perfusion imaging in the detection of functionally significant obstructive luminal stenoses in patients with known or suspected CAD in comparison to FFR. The secondary objectives are to investigate the diagnostic accuracy of CMR in the diagnosis of haemodynamically obstructive CAD on the basis of magnetic flux density (defined in Tesla) and basis of analysis (coronary artery territory *vs.* patient basis). We will also be able to assess the methodological quality of the included studies.

### Statistical analysis and data synthesis

Data analysis will be performed according to the following steps: (1) inter-rater reliability testing for study selection and assessment of methodological quality; (2) calculation of test accuracy measures (sensitivity, specificity and likelihood ratios); (3) testing of heterogeneity; (4) pooling of data; and (5) sub-group analysis.

Agreement between the reviewers (inter-rater reliability) in evaluating methodological quality by using the revised QUADAS-2 tool will be calculated with Cohen Kappa coefficient (*k*) [20]. QUADAS-2 suggested tabulations [19] will also be used to present assessment results of overall performance of the selected studies. We will also calculate the sensitivity, specificity, positive and negative likelihood ratio (LR) and the diagnostic odds ratio (DOR) along with 95% confidence intervals (CI) to determine the diagnostic accuracy of each index test in differentiating non-significant from significant and haemodynamically critical coronary arterial luminal stenosis.

To statistically determine the heterogeneity in the studies we will use Cochrane Q (*x*^*2*^ statistic) test and the *I*^*2*^ statistic for heterogeneity. Evidence of clinically relevant heterogeneity will be considered when the *I*^*2*^ statistic is >25% [21]. In cases where clinically relevant heterogeneity is found, outcome measures will be estimated from studies factoring the highest scientific validity (studies that are most likely to be free from bias). Using bivariate random effects model (BREM), pooled and individual estimates of sensitivity and specificity at 95% CI will be presented in forest-plot diagrams. We will also generate summary of receiver operator curves (SROC) using point estimates of each study as well as symmetrical summary curve, a summary point estimate, 95% confidence region and 95% prediction region using STATA version 13 (xtmelogit command) as it has the capabilities [22]. We also intend to use multivariate random effects model in the calculation pooled analysis of CMR’s diagnostic accuracy in relation to different FFR thresholds [23]. Direct comparison of CMR and FFR will also be achieved by using bivariate models.

We will evaluate the presence of possible publication bias graphically by drawing funnel plots for each outcome measure and statistically by means of Egger’s standard regression test. Two-tailed *P* <0.10 tests will be assumed to be statistically significant. We will also acknowledge methodological heterogeneity or true study heterogeneity as these could also introduce asymmetry in publication bias assessment [24]. The bivariate random effects model was preferred for this protocol as it preserves the two-dimensional nature of the original data. It also allows the estimation of between study variation in sensitivity and specificity separately in addition to the degree of correlation between the two [25].

## Discussion

The assessment of the haemodynamic clinical relevance of coronary arterial lesions seen on routine qualitative coronary angiography is important as evidence has emerged showing no long-term symptomatic or survival benefit for routine revascularisation procedures in patients with stable coronary disease [26]. Conversely, there is evidence for improved outcomes following revascularisation in subjects with known haemodynamically significant coronary artery lesions [3-6]. Previous diagnostic test studies comparing CMR and FFR have reported variable results. Whether this is due to imprecision in reporting or variable methodological quality it is still not clear and needs to be explored. This systematic review will allow determination as to whether CMR is an adequate screening test in the diagnosis of significant obstructive CAD. It will also determine if CMR could replace FFR for diagnosing coronary arterial lesions that are haemodynamically significant, and allow consideration of the potential benefit of CMR in diagnosing ischaemic [27] and non-ischaemic myocardial pathologies [28-30] without exposing patients to ionising radiation. This systematic review may also identify the type of CMR magnetic field strength that could provide the greatest sensitivity and specificity. The results of the review will be communicated as abstract presentations at conferences and will be published in a peer-reviewed journal.

## Abbreviations

AUC: Area under the curve; CAD: Coronary artery disease; CMR: Cardiac magnetic resonance; FFR: Fractional flow reserve; LR: Likelihood ratio; SROC: Summary of receiver operator curves; QUADAS: Quality assessment of diagnostic accuracy studies.

## Competing interests

The authors declare that they have no competing interests.

## Authors’ contributions

RBP was responsible for drafting, designing, revision and editing of the manuscript. MM, TK and MN were responsible for the design and conception of the study. MC and FW were responsible for extensive final revision of the manuscript including grammar. All the authors read and approved the manuscript.

## Supplementary Material

Additional file 1 The search strategy for OVID MedLine. Click here for file

Additional file 2 The search strategy used in PubMed Database. Click here for file

Additional file 3 Risk of bias and applicability judgements in QUADAS-2. Click here for file
