# Supplementary material for: Magnetic resonance myocardial perfusion imaging in the diagnosis of functionally significant obstructive coronary artery disease: a systematic review protocol
Source: Syst Rev. 2014 May 26;3:53. doi: 10.1186/2046-4053-3-53 (PMC4048601; doi:10.1186/2046-4053-3-53)
Supplement: Additional file 1 — The search strategy for OVID MedLine. [file 2046-4053-3-53-S1.doc]

**Additional file 1: Showing the** search strategy for OVID MedLine

1. Cardiac

2. Cardiovascular

3. Magnetic resonance perfusion imaging

4. CMR

5. cmr

6. CMR-MPI

7. 1 OR 2 OR 3 OR 4 OR 5 OR 6

8. FFR

9. Fractional flow reserve

10. Pressure- wire guided fractional flow reserve

11. Coronary angiography guided fractional flow reserve

12. 8 OR 9 OR 10 OR 11

13. 7 AND 12

14. Significant obstructive coronary artery disease

15. Coronary artery disease

16. CAD

17. Percutaneous coronary intervention AND fractional flow reserve

18. Pressure-wire guided FFR AND cardiac magnetic resonance perfusion imaging

19. 14 OR 15 OR 16 OR 17 OR 18

20. 13 AND 19

20. Sensitivity

21. Specificity

22. False positive

23. False negative

24. Detection

25. Diagnostic

26. Accuracy of CMR perfusion

27. 20 OR 21 OR 22 OR 23 OR 24 0R 25 OR 25 AND 26

28. 20 AND 27
