# Supplementary material for: Magnetic resonance myocardial perfusion imaging in the diagnosis of functionally significant obstructive coronary artery disease: a systematic review protocol
Source: Syst Rev. 2014 May 26;3:53. doi: 10.1186/2046-4053-3-53 (PMC4048601; doi:10.1186/2046-4053-3-53)
Supplement: Additional file 2 — The search strategy used in PubMed Database. [file 2046-4053-3-53-S2.doc]

| **Additional file 2** : **Showing the search strategy used in PubMed Database** |
| --- |
| 1. "cardiovascular system"[MeSH Terms] OR ("cardiovascular"[All Fields] AND "system"[All Fields]) OR "cardiovascular system"[All Fields] OR "cardiovascular"[All Fields] 2. "heart"[MeSH Terms] OR "heart"[All Fields] OR "cardiac"[All Fields] 3. "magnetic resonance imaging"[MeSH Terms] OR ("magnetic"[All Fields] AND "resonance"[All Fields] AND "imaging"[All Fields]) OR "magnetic resonance imaging"[All Fields] 4. "perfusion imaging"[MeSH Terms] OR ("perfusion"[All Fields] AND "imaging"[All Fields]) OR "perfusion imaging"[All Fields] 5. ("heart"[MeSH Terms] OR "heart"[All Fields] OR "cardiac"[All Fields]) AND ("perfusion"[MeSH Terms] OR "perfusion"[All Fields]) AND ("magnetic resonance imaging"[MeSH Terms] OR ("magnetic"[All Fields] AND "resonance"[All Fields] AND "imaging"[All Fields]) OR "magnetic resonance imaging"[All Fields] OR "mri"[All Fields]) 6. #1 OR #2 OR #3 OR #4 OR #5 7. fractional[All Fields] 8. flow[All Fields] AND reserve[All Fields] 9. Fractional[All Fields] AND flow[All Fields] AND reserve[All Fields] 10. fractional[All Fields] AND flow[All Fields] AND reserve[All Fields] AND versus[All Fields] AND ("angiography"[MeSH Terms] OR "angiography"[All Fields]) 11. fractional[All Fields] AND flow[All Fields] AND reserve[All Fields] AND guided[All Fields] 12. fractional[All Fields] AND flow[All Fields] AND reserve-guided[All Fields] 13. fractional[All Fields] AND flow[All Fields] AND reserve[All Fields] AND ("heart"[MeSH Terms] OR "heart"[All Fields] OR "coronary"[All Fields]) 14. fractional[All Fields] AND flow[All Fields] AND reserve[All Fields] AND guided[All Fields] AND pci[All Fields] 15. ffr[All Fields] AND guided[All Fields] AND pci[All Fields] 16. ffr[All Fields] AND ("heart"[MeSH Terms] OR "heart"[All Fields] OR "coronary"[All Fields] 17. #7 OR #8 OR #9 OR #10 OR #11 OR #12 OR #13 OR #14 OR #15 OR #16 18. 6 AND #17 |
