# Supplementary material for: Magnetic resonance myocardial perfusion imaging in the diagnosis of functionally significant obstructive coronary artery disease: a systematic review protocol
Source: Syst Rev. 2014 May 26;3:53. doi: 10.1186/2046-4053-3-53 (PMC4048601; doi:10.1186/2046-4053-3-53)
Supplement: Additional file 3 — Risk of bias and applicability judgements in QUADAS-2. [file 2046-4053-3-53-S3.doc]

| **Additional file 3: Risk of bias and applicability Judgments in QUADAS-2** | | | | |
| --- | --- | --- | --- | --- |
| **Domain** | **Patient selection** | **Index test**  *Cardiac magnetic Resonance perfusion imaging*  *(CMR)* | **Reference** *standard*  *Fractional flow reserve(FFR)* | **Flow and timing** |
| Description | Methods of patient selection are described.  Patients included are described (if had previous testing, how they presented, if intended to use index test, and setting) | Index test is described and how it was conducted and interpreted) | Reference standard is described including how it was conducted and interpreted | Any patients who did not receive the index tests or reference standard or excluded from 2 by 2 table are descrided  The interval any interventions between index tests and the reference standard are should be described |
| Signalling questions ( yes, no, unclear) | Does the study include consecutive or randomly enrolled patients?  Did it avoid case-control design?  Did the study avoid inappropriate exclusion? | Were the index test results interpreted without the knowledge of the reference standard?  Did the authors use a prespecified threshold , if used? | Is the reference standard likely to correctly classify the target condition?  Were the reference standard results results interpreted without knowledge of the results of the index test? | Was there an appropriate interval between index tests and the reference standard?  Did all the patients receive a standard reference?  Did all the patients receive the same reference standard?  Were all the patients included in the analysis? |
| Risk of bias (high, low or unclear) | Could the selection of patients have introduced bias | Could the conduction or interpretation of the index test have introduced bias | Could the reference standard, its conduct, or its interpretation have introduced bias? | Could the patient flow have introduced bias? |
| Concerns about applicability ( high, low or unclear) | Are there concerns that the included patients do not match the review question | Are there concerns that the index test, its conduct, or its interpretation differ from the review question? | Are there concerns that the target condition are defined by the reference standard does not match the review question? |  |

Adapted from Annals of internal medicine; research and reporting methods

By Whiting *etal* QUADAS-2: A Revised Tool for the Quality Assessment of Diagnostic Accuracy Studies;2011; Ref: Ann Intern Med. 2011;155(8):529-536. doi:10.7326/0003-4819-155-8-201110180-00009
